# Inflammatory Bowel Disease Treatments and Predictive Biomarkers of Therapeutic Response

**DOI:** 10.3390/ijms23136966

**Published:** 2022-06-23

**Authors:** Duaa Ahmed Elhag, Manoj Kumar, Marwa Saadaoui, Anthony K. Akobeng, Fatma Al-Mudahka, Mamoun Elawad, Souhaila Al Khodor

**Affiliations:** 1 Research Department, Sidra Medicine, Doha 26999, Qatar; delhag@sidra.org (D.A.E.); mkumar@sidra.org (M.K.); msaadaoui@sidra.org (M.S.); 2 Division of Gastroenterology, Hepatology and Nutrition, Sidra Medicine, Doha 26999, Qatar; aakobeng@sidra.org (A.K.A.); falmudahka@sidra.org (F.A.-M.); melawad@sidra.org (M.E.)

**Keywords:** IBD, precision medicine, Crohn’s disease, ulcerative colitis, biomarkers, biological treatment

## Abstract

Inflammatory bowel disease (IBD) is a chronic immune-mediated inflammation of the gastrointestinal tract with a highly heterogeneous presentation. It has a relapsing and remitting clinical course that necessitates lifelong monitoring and treatment. Although the availability of a variety of effective therapeutic options including immunomodulators and biologics (such as TNF, CAM inhibitors) has led to a paradigm shift in the treatment outcomes and clinical management of IBD patients, some patients still either fail to respond or lose their responsiveness to therapy over time. Therefore, according to the recent Selecting Therapeutic Targets in Inflammatory Bowel Disease (STRIDE-II) recommendations, continuous disease monitoring from symptomatic relief to endoscopic healing along with short- and long-term therapeutic responses are critical for providing IBD patients with a tailored therapy algorithm. Moreover, considering the high unmet need for novel therapeutic approaches for IBD patients, various new modulators of cytokine signaling events (for example, JAK/TYK inhibitors), inhibitors of cytokines (for example IL-12/IL-23, IL-22, IL-36, and IL-6 inhibitors), anti-adhesion and migration strategies (for example, β7 integrin, sphingosine 1-phosphate receptors, and stem cells), as well as microbial-based therapeutics to decolonize the bed buds (for example, fecal microbiota transplantation and bacterial inhibitors) are currently being evaluated in different phases of controlled clinical trials. This review aims to offer a comprehensive overview of available treatment options and emerging therapeutic approaches for IBD patients. Furthermore, predictive biomarkers for monitoring the therapeutic response to different IBD therapies are also discussed.

## 1. Introduction

Inflammatory bowel disease (IBD) is a chronic relapsing inflammatory disorder of the gastrointestinal (GI) tract [1]. Multiple factors including urbanization, westernization, dietary changes, increased antimicrobial exposure, and other factors affecting host–microbial homeostasis have been linked to an increase in the prevalence of IBD [2]. IBD is a chronic disease that causes progressive structural and functional damage to the GI tract and intestinal epithelium [3] requiring lifelong medication [1]. IBD is classified into two major subtypes based on pathological features and disease manifestation: Ulcerative Colitis (UC), which primarily affects the colon, and Crohn’s disease (CD), which affects multiple GI sites, suggesting that these subtypes are distinct clinical entities that require distinct clinical management [4,5]. CD and UC are considered highly heterogeneous and complex, which further complicates the clinical management and treatment plans for those patients [5].

A better understanding of disease biology and heterogeneity has resulted in the development of broad-spectrum and disease-specific molecules employed for precise targeting, resulting in a major improvement in therapy effectiveness and outcomes [6]. Though developing treat-to-target techniques has improved IBD patients’ quality of life, we still face a considerable therapeutic ceiling [7], since a significant proportion of patients either do not react to therapy or lose response over time [8]. Although the mechanisms driving the lower efficacy of IBD medications are unknown, the ability to anticipate treatment response would allow patients with refractory conditions to receive individualized treatment options. This review will discuss several newly approved and impending IBD therapeutic options, as well as offer a literature review on predictive biomarkers of therapeutic response to various IBD treatments.

## 2. Disease Classification, Activity and Severity Assessment Tools

IBD has historically been subclassified into two subtypes CD and UC, though it is a highly heterogeneous condition; therefore, its disease spectrum and complexity cannot be explained by a single CD or UC phenotype. The disease spectrum of IBD is affected by multiple factors such as age of onset of disease, genetic background, microbiome, dietary habits, clinical aspects and disease location classification (for example small bowel-predominant CD is different from colonic predominant CD or left sided UC is different from extensive UC that progressed), disease granularity (rectal involvement or colonic extension) and disease behavior (fibrosing or penetrating) [5]. Besides the disease complexity of IBD subtypes, some other pathologies can also mimic IBD-like disease such as intestinal Behçet, Mediterranean fever enterocolitis, and other microbial infectious causes (including Entamoeba) [5]. The IBD heterogenicity and complexity can significantly influence the treatment outcomes and clinical management of patients. For example, up to 30% of patients do not respond to initial therapy and even among initial responders, 13–46% lose response over time with estimates varying by treatment and disease subtypes [9], a percentage that can sometimes reach as high as 64% after treatment [10]. Therefore, a periodic assessment of IBD activity and disease severity is required to assess disease phenotype, including disease extent and severity in UC, as well as disease extent and disease behavior in CD, to provide a tailored therapy algorithm to every patient [5,11,12,13].

Disease activity in IBD patients is evaluated by combining multiple invasive and/or non-invasive procedures such as patient-reported symptoms, inflammatory markers score, endoscopic assessment, capsule endoscopy, single- or double- balloon enteroscopy, MRI scores, and histology scores [8,14,15,16,17,18,19,20,21,22]. Endoscopic assessment of the gastrointestinal tract is known to be the gold standard method for assessing disease activity, and it has a good correlation with serological markers; however, because endoscopic assessment is an invasive method, it cannot be performed routinely to monitor disease severity [23,24,25,26,27,28,29,30,31]. As a result, non-invasive IBD activity markers, such as fecal markers and serological markers, are advantageous for monitoring disease severity. Table 1 summarizes the various methods used to track disease activity in IBD patients. To grade disease activity, these methods combine patient-reported symptoms (such as the number of stools per day, abdominal pain, and rectal bleeding) with extraintestinal manifestations, physical examination findings, endoscopy results, and hematocrit [32,33,34,35,36,37,38].

## 3. Treatment Options for CD and UC

**Figure 1:**
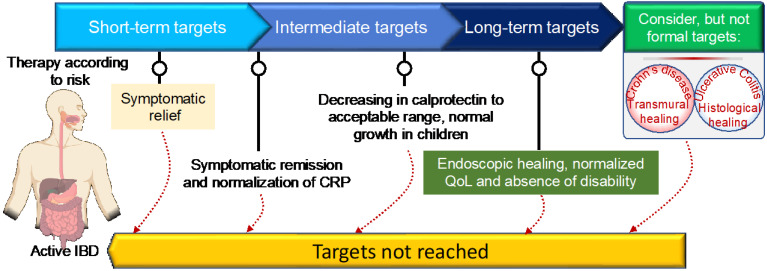
STRIDE-II recommendations for disease monitoring and clinical management of inflammatory bowel disease using short- and long-term target goals.

IBD has no known cure. Based on recent treatment strategies, the Selecting Therapeutic Targets in Inflammatory Bowel Disease (STRIDE)-II encompasses evidence-based recommendations for IBD patients [39]. The first short-term target of IBD treatment is to control the acute GI inflammation that causes signs and symptoms, which usually results in not only symptom relief but also long-term symptomatic remission and normalizing CRP to reduce further complications. Currently, IBD management has been centered on symptomatic response and endoscopic healing, with four main goals: [1] symptomatic relief, defined as an immediate goal, acknowledging that this is rated highest by patients; [2] symptomatic remission and normalization of CRP, defined as preventing disease flare-ups; [3] decreasing calprotectin and improving the patient’s quality of life and normal growth; and [4] Endoscopic healing with clinical remission in absence of disability. In addition, transmural healing in CD patients and histological healing in UC patients are newly recommended adjunctive measures of the depth of treatment response but are not yet endorsed as formal new treatment targets [39]. Although oral aminosalicylates and corticosteroids are highly effective in suppressing acute GI inflammation, resolving symptoms, and inducing remission, they are unable to reduce long-term complications, improve the patient’s long-term outcomes, or promote healing after mucosal damage. As a result of recent biologic therapy breakthroughs, STRIDE-II encompasses evidence-based recommendations for a paradigm shift in the clinical management of IBD patients, with an emphasis on long-term targets of clinical remission and endoscopic healing in absence of disability, and a restoration of quality of life and normal growth in children [39]. Figure 1 depicts the current STRIDE-II recommendations for therapeutic monitoring of IBD management. The IBD medications fall into the following basic categories:

### 3.1. Aminosalicylates

**Figure 2:**
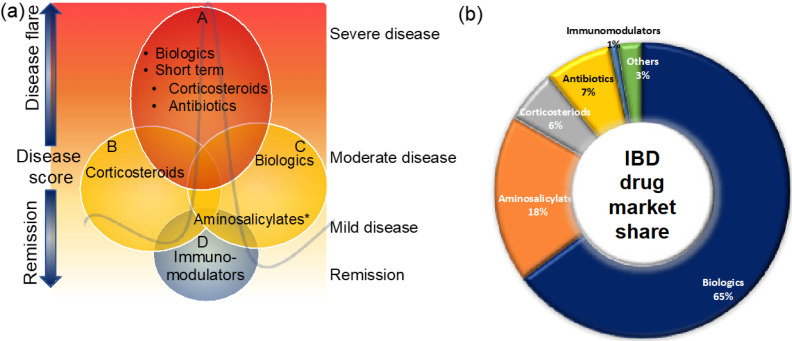
Clinical management of IBD patients during disease flare and remission (**a**) and the market share of IBD medicines (**b**). Maintaining remission and prevention of disease flare that triggers signs and symptoms is the main goal of IBD treatment. This figure gives an overview of the current clinical management of IBD patients. For more details, see the main text. * Some aminosalicylates such as balsalazide and mesalamine are approved for mild-to-moderate UC patients.

These therapies are small molecules that are administered orally or rectally to decrease the inner wall inflammation of the intestines (Figure 2). Aminosalicylates are known to be the first-line treatment option for UC patients with mild-to-moderate disease and the second most prescribed IBD medicine [40,41,42] (Figure 2a,b). Aminosalicylates have a wide range of anti-inflammatory and immunomodulatory functions, including inhibition of cyclooxygenase, lipoxygenase, platelets-activating factor, interleukin (IL)-1 nuclear factor B, and scavenging of reactive oxygen species [43,44,45]. Emerging evidence suggests that aminosalicylates keep IBD patients in remission by preventing leukocyte recruitment into the bowel wall [46,47].

### 3.2. Corticosteroids

Corticosteroids are non-selective systemic anti-inflammatory therapies that can be given orally, rectally, or intravenously and are very effective for short-term treatment of moderate-to-severe CD and UC patients [48]. Corticosteroids mediate their immunosuppressive effects by reducing the aberrant production of cytokines such as IL-1, IL-2, IL-3, IL-4, IL-5, IL-6, IL-8, IL-10, IL-12, TNF-α, IFN-γ, and GM-CSF, according to the mechanism of action studies. [49,50]. The reduced synthesis of proinflammatory cytokines helps in the induction of remission in patients with active IBD. However, their long-term treatment is not recommended due to significant adverse effects such as an increased risk of mortality, infection [51], osteoporosis, psychological disturbances including insomnia, schizophrenia, depression, and euphoria, moon face, fat deposition, dermatological disorders, steroid-induced diabetes [52] and a negative effect on growth in prepubescent children.

Given the high clinical demand, many second-generation corticosteroids with improved safety profiles for the clinical management of IBD have emerged in the last two decades (Table 2). Although corticosteroids are very effective at controlling short-term inflammation in IBD patients, they are ineffective at achieving endoscopic remission or healing the mucosa in both UC and CD patients [50,53].

### 3.3. Immunomodulators

Immunomodulator therapies are administered orally or intravenously to patients to modulate their immune systems and reduce inflammation. Typically, immunomodulators are effective in maintaining remission and are prescribed to patients who are not responding to aminosalicylates and corticosteroids, or as adjuvant treatment to anti-TNF to prevent anti-body formation, particularly with infliximab [54] or as adjuvant treatment to anti-TNF to prevent antibody formation particularly with infliximab [55]. The MOA of different immunomodulators is summarized in Table 2.

### 3.4. Antibiotics

The long-term intestinal inflammation in IBD patients is often associated with gut microbial dysbiosis or intra-abdominal infections [2,56]. In addition, CD is usually associated with abscesses (pockets of pus) or fistulae (connection of diseased bowel to other body part such as bladder, skin, another bowel piece or vagina, which are usually associated with bacterial infections [57]). These microbial infections can mimic the symptoms of an IBD flare. Manipulating the gut microbiota or intestinal infections can be achieved by prebiotics (dietary therapies), fecal transplants (discussed below) and antibiotics. The British Society of Gastroenterology (BSG) recommends the important role of antibiotics for treating secondary complications in CD such as abscesses and bacterial overgrowth [58] and the European Crohn’s and Colitis Organization (ECCO) guidelines recommend the use of antibiotics in case of an acute infection or prior to surgery in UC patients [59]. Therefore, antibiotics are often prescribed for managing IBD patients (including luminal and fistulizing disease for CD and colitis in the case of UC), for treating bacterial infections, or for septic complications of IBD, such as abscesses and post-surgery to prevent disease recurrence [60] (Table 1). Antibiotics may also be used to maintain remissions, or for the treatment of pouchitis [61]. Normally antibiotics are a short-term treatment for IBD patients.

### 3.5. Biologic Therapies

Because many IBD patients do not respond to standard anti-inflammatory and immune modulator medications, there has been a clear need for more specific novel therapeutic approaches to be developed. Bioengineered antibodies that target specific molecules or proteins that cause inflammation or are involved in the inflammatory process are known as biologic therapies [62,63]. Biological therapies are typically prescribed to patients who have moderate-to-severely active disease and have not responded well to conventional therapy [62] (Figure 2). Biologics therapies may be an effective strategy for reducing long-term steroid use as well as maintaining remission; this could be one of the reasons biologics have captured the largest share of the IBD market (Figure 2b). In recent years, there has been a growing trend toward using biologic therapy as first-line therapy in certain clinical situations [64].

#### 3.5.1. Specific Treatment Options for CD and UC: Treat-To-Target Approach

Cytokines appear to play a significant role in driving intestinal, systemic, and extra-intestinal inflammation in IBD patients. Targeting pro-inflammatory cytokines such as TNF and other distinct cytokines produced by APCs has already been shown to be effective in suppressing chronic intestinal inflammation, implying that cytokine blockade or targeting cytokine signaling cascades are important fields of interest for clinical management of IBD.

#### 3.5.2. TNF-Inhibitors

Given the importance of tumor necrosis factor (TNF) in the pathogenesis of IBD, several TNF-inhibitors have been developed to control intestinal inflammation and the clinical symptoms of IBD (Table 2). TNF-α plays such an important role that anti-TNF agents such as adalimumab, infliximab, certolizumab, and golimumab are now used as standard-of-care therapy for both UC and CD management [65,66]. Interestingly, infliximab has been shown effective in moderate-to-severe UC and CD patients for inducing and maintaining remission, with transmural healing in CD and histological healing in UC, suggesting the broad relevance of anti-TNF-therapy [67]. During intestinal inflammation, TNF is produced by various immune cells including macrophages, T-cells and dendritic cells in the gut of IBD patients [68], to induce neo-angiogenesis [69], activate various mucosal immune cells to produce pro-inflammatory cytokines, and stimulate Paneth cell death via necroptosis [70] or by inducing apoptosis of intestinal epithelial cells [71]. Thus, TNF inhibition can suppress intestinal inflammation through a variety of mechanisms. Recognizing the significant potential of anti-TNF therapies in the treatment of IBD, several biosimilars of TNF-inhibitors have been developed and approved by the Food and Drug Administration (FDA), including adalimumab biosimilars-Hyrimoz™ (adalimumab-adaz), Cyltezo™ (adalimumab-adbm), Amjevita™(adalimumab-atto), infliximab biosimilar-Ixifi™ (infliximab-qbtx), Renflexis™(infliximab-abda), Inflectra™(infliximab-dyyb) [72].

#### 3.5.3. CAM Inhibitors

Clinical management of IBD patients has revealed that 30–50 percent of patients either do not respond to anti-TNF therapy or have decreased efficacy over time, implying the need for new alternative therapies [73]. Emerging experimental studies have indicated that inhibitions of activated cell adhesion molecule (CAM) in the inflamed intestinal tissue might provide a new therapeutic option for intestinal inflammation [74]. Natalizumab, the first anti-CAM antibody, was later approved for the treatment of CD patients. Natalizumab has demonstrated significant clinical efficacy in moderate-to-severe CD patients by inhibiting lymphocyte trafficking into the gut via binding to 4-integrins, a ligand known to play an important role in the recruitment of T-cells to intestinal tissues and cause intestinal inflammation [75]. The clinical efficacy was mediated by inhibiting the interaction between α4β7 in the gut and the α4β1 in the blood brain barrier with their ligands (VCAM1 and MAdCAM1, respectively), affecting the homing of immune cells across the gut endothelium and blood–brain barrier, respectively [76,77]. However, despite potent clinical efficacy, long-term natalizumab treatment resulted in a rare but lethal John Cunningham virus (JCV) infection [77,78]. The JCV infection was probably associated with the nonspecific binding mechanism of natalizumab [77,78], highlighting the need for a more specific blockade of α4β7-integrins. Following that, more specific monoclonal IgG antibodies, such as vedolizumab, were developed for moderate-to-severe UC (Table 2), and a few more are currently in clinical trials. Vedolizumab is a novel monoclonal IgG1 antibody that inhibits lymphocyte trafficking into the gut while not interfering with the blood–brain barrier [79,80]. The efficacy of vedolizumab is mediated through the selective blocking of lymphocyte binding to α4β7 integrin in patients with moderate-to-severe IBD [79,80]. The specific inhibition of β7 integrin has been shown to lower the incidence of systemic side effects and to induce long term clinical remission [81,82]. Considering the success of the anti-α4β7 integrin approach, emerging therapies targeting T-cell homing such as etrolizumab, a selective inhibitor of both α4β7 and αEβ7 integrins and ontamalimab, a selective binding inhibitor of MAdCAM-1 to the α4β7 ligand, are the emerging new monoclonal IgG1 and IgG2 antibodies for moderate-to-severe UC and CD patients [79]. AJM300 is another orally active humanized anti-α4 integrin antagonist, inhibits the binding of α4β1 with VCAM-1 and α4β7 with MAdCAM [83] in clinical development for UC patients.

#### 3.5.4. Anti-Interleukin Inhibitors

Ustekinumab is a newly approved biologic treatment that targets the p40 subunit of interleukin-12 (IL-12) and IL-23 which are proinflammatory cytokines that play a role in the pathogenesis of IBD [84,85]. It has been approved by FDA for the treatment of adult IBD patients with moderate-to-severe disease. Ustekinumab has shown effectiveness in inducing and maintaining clinical remission in active CD and UC patients [85,86]. Risankizumab is another humanized monoclonal IgG1 antibody that targets the p19 subunit of IL-23 in clinical development. IL-23 is known to play a substantial role in the regulation of the T-helper 17 cells and stimulation of pro-inflammatory cytokines in IBD patients [87]. Preliminary clinical trial results indicate that Risankizumab is well tolerated and able to mediate long-term clinical response and endoscopic remission in active CD patients [88].

### 3.6. JAK Inhibitors

Following the success of biologics in the clinical management of IBD patients, there has been intensive research for alternative effective anti-cytokine strategies. Tofacitinib (CP-690,550) is the first-in-class, oral, pan-Janus kinase (JAK) inhibitor known to be effective and safe for moderate-to-severe UC patients [89] (Table 2). MOA studies reveal that Tofacitinib inhibits JAK-1, JAK-2, and JAK-3 and thereby blocks the signaling pathway of gamma chain-containing cytokines, mainly IL-2, IL-4, IL-7, IL-9, IL-15, and IL-21. Interestingly, JAK inhibition has been found to be effective in suppressing T-cells, natural-killer cells, and modulating proinflammatory cytokines; something which has opened the possibility of blocking the activity of several proinflammatory cytokines simultaneously [90]. Indeed, various JAK inhibitors filgotinib (formerly called GLPG0634, GS-6034), PF-06651600, TD-1473, etc., are being evaluated in different clinical trials. Although preliminary clinical results suggest efficacy in moderate-to-severe IBD patients, their safety profiles must be determined in larger phase III clinical trials.

### 3.7. Dietary Therapies

The link between dietary intake and intestinal inflammation has substantially altered our preference for dietary changes in the clinical management of IBD [91]. Dietary intake may facilitate intestinal inflammation through various mechanisms including modulating the gut microbiome, tight junctions, and mucous layer [92]. Therefore, various dietary therapies, such as exclusive enteral nutrition (EEN) and CD exclusion diet etc., have been explored in recent years for their potent therapeutic role in the management of IBD patients.

EEN is the most widely studied and replicated dietary intervention for CD patients, including pediatric patients, with primary outcomes focusing on induction of clinical remission and mucosal healing [93,94]. Multiple emerging studies indicate that EEN mediates therapeutic effects through modulation of the gut microbiota, by affecting the gut permeability, and by stimulating the immune system, which in-term might lead to endoscopic remission in patients with mild-to-moderate CD [91,95]. Although EEN can help in controlling intestinal inflammation by avoiding the potentially harmful dietary components, the exclusive character of EEN, in which either exclusive or partial formula-based diets are used, is still controversial [96]. Based on the EEN data, more tolerable but still effective solid foods have been explored, such as the new CD exclusion diet (CDED) [97], CD TReatment-with-EATing (CD-TREAT) [98], the specific carbohydrate diet (SCD) [99] and, interestingly, these data revealed the first promising results, emphasizing the role of diet in controlling inflammation in patients with CD by excluding specific food ingredients (94). These dietary interventions incorporate a large amount of high-quality protein, minimize fat content, and incorporate food items rich in complex carbohydrates including natural foods such as chicken, eggs, potatoes, rice, fruits, and vegetables, to assure the patient’s lean mass growth and restoration [100]. Although these dietary-based treatments are more executable compared to EEN, they still need a strict attachment to the protocols, constraining their adherence over time.

Recognizing the potential therapeutic role of dietary therapies in IBD, a plethora of new dietary intervention strategies are currently being explored in clinical trials in IBD that may challenge established treatment regimens in future. For examples, two recent CDED clinical trials on pediatric and adult CD patients identified the effectiveness of both CDED and the partial enteral nutrition (PEN) in inducing remission in individuals with mild-to-moderate CD compared to EEN diet (NCT01728870, NCT02231814) [94,97]. The preliminary results from other dietary based treatments including the specific carbohydrate diet (SCD) or Mediterranean diet (MD) revealed significant clinical and mucosal improvements in IBD patients through a promotion of the gut microbiome and metabolomes associated with remission and lowering the levels of fecal calprotectin [97,101,102]. Interestingly, more promising studies are now investigating the role of nutritional interventions in combination with analyses of gut microbiome and metabolome, aiming to restore the healthy gut microbiome balance and providing a new hope for individuals with IBD (NCT04018040, NCT04552158, NCT02858557).

## 4. Emerging Therapies for CD and UC

### 4.1. Sphingosine-1-Phosphate Receptor

The discovery of Sphingosine-1-phosphate (S1P) receptor inhibitors is another significant advancement in the modulation of immune cell trafficking for IBD clinical management. Ozanimod and Etrasimod are novel orally administered small molecules with potent and selective S1P receptor agonist activity. The S1P receptor has five subtypes: S1P 1–5, and it plays an important role in the regulation of many physiological and pathophysiological processes, such as NF-kB, STAT3 transcription factors, angiogenesis, cancer, cellular inflammation through cellular proliferation, and intracellular communication via lymphocyte trafficking to lymphoid organs and circulation [103]. Ozanimod specifically binds to S1P 1 and 5 receptors, whereas Etrasimod binds to the S1P receptor, with both molecules being currently tested in randomized clinical trials against moderate to severe UC patients (Table 3) [104,105]. Although preliminary clinical efficacy data for both drugs in moderate-to-severe UC patients showed a significant clinical response with a higher clinical remission rate, with mucosal healing and histological better remission compared with a placebo [104,106], their adverse effects include anemia, exacerbation of UC in some patients and headaches [106]. Additional long-term studies are currently underway to assess their potency and safety in moderate-to-severe UC (NCT03915769, NCT03945188).

### 4.2. Stem-Cell Therapies

Emerging evidence suggests that stem-cell therapies, by modulating the mucosal immune response, could be used as an alternative method to treat inflamed tissue damage [107]. Hematopoietic stem cells (HSCs) and mesenchymal stem cells (MSCs) are multipotent cells derived from bone marrow, umbilical cord, and adipose tissue, respectively. Both therapies are being studied for their immunomodulatory properties in CD and UC patients, to downregulate aberrant mucosal immune responses and promote regulatory T-cell formation and tissue healing [108,109]. Interesting preliminary results of Cx-601 (MSCs) and HSC transplants have shown efficacy in inducing clinical remission and endoscopic healing in CD patients [110,111]; however, their results were inconsistent and even associated with adverse events, mainly infection [112]. Despite clinical inconsistencies, Cx-601 is being evaluated in the phase-III trial for long-term benefits (NCT03706456). Reports from pediatric HSC transplants have shown promising results in very early inflammatory bowel disease with fewer complications using allogeneic reduced-intensity conditioning, particularly in IL-10 and receptor deficiency [113].

### 4.3. Antisense Nucleotide

Mongersen is a small antisense nucleotide that inhibits the translation of SMAD7, a TGF-β signaling protein (Table 3). Despite encouraging efficacy data in CD patients [114], its clinical development was halted due to a lack of consistency in the results [115].

### 4.4. Microbial-Based Therapeutics: To Decolonize the Bed Buds

The emerging results from microbiome research indicate that micro-organisms are an intrinsic part of the human body, affecting all aspects of life [116,117,118,119], and have inspired exploration of their role in the IBD [2]. Gut microbiota of IBD patients has revealed a decrease in microbial diversity, as evidenced by lower numbers of Firmicutes, Bacteroides, and Actinobacteria and higher numbers of Enterobacteriaceae [120]. Growing evidence indicates that microbial dysbiosis has been a hallmark of the IBD pathophysiology [2].

### 4.5. Fecal Microbiota Transplantation

Considering the importance of microbial diversity in maintaining gut homeostasis, certain approaches such as fecal microbiota transplantation (FMT) have received considerable attention in recent years. FMT is a process of re-establishing a healthy gut microbiome by limiting the colonization of certain species while promoting the growth of others by infusing a fecal inoculation from a healthy donor into the GI tract of a recipient patient [121]. Although the specific mechanism of FMT success remains unknown, it has shown promising results in treating *Clostridium difficile* infection [122,123]. Given the overlap of gut microbial dysbiosis between CD and UC, FMT is being extended for evaluation as a new therapy in IBD. There are currently 55 FMT clinical studies for different bowel diseases, including 20 for CD and 18 for patients with UC (https://clinicaltrials.gov (accessed on 7 June 2022).

FMT is often performed in patients with relatively low α-diversity [124], which may facilitate the engraftment of healthy microbiota [124,125]. Although active research for FMT is being conducted, the lack of consistency in efficacy in IBD patients necessitates more research to identify the ideal microbiota composition to induce long-term efficacy of FMT. Although new research shows a clear link between gut microbiota and IBD, no single pathogen has been identified as the causative agent [2]. In addition to the low efficacy of FMT, other challenges include the risk of transferring pathogenic strains, lack of standardized procedures, and unwanted induction of flares in some UC patients [126]. As a result, it is ironic that, at a time of rapid technological advances in metagenomics and computational tools that have increased our understanding of the gut microbiota, FMT is likely to be replaced by the use of defined microbial consortia. Future research will be needed to optimize the microbial composition, and delivery aspect, and reduce the possibility of pathobionts transmission.

### 4.6. Bacterial Inhibitor

IBD may be driven by the presence of persistent pathogens (such as members of *Enterobacteriaceae*) that can adapt to an oxidizing hostile environment and exacerbate the disease pathogenesis [2]. In this context, members of the phylum *Enterobacteriaceae*, specifically *Escherichia coli*, are frequently reported at higher abundance in CD patients [127]. Emerging technologies in microbiome therapeutics have made it possible to selectively remove specific microbes to control microbial outgrowth and modulate gut microbial homeostasis [128]. The adherent-invasive *E. coli* (AIEC) strains can adhere to the small bowel epithelium in ileal mucosa using the *FimH* gene [129] and may represent a viable target for such emerging approaches. Phage therapy and antagonists of the *FimH* receptor can inhibit the AIEC strains or their attachment to epithelial cells and this holds great promise in emerging microbiome therapeutics. Although the preliminary results are encouraging [130,131], we must wait for ongoing phase II trials of EB8018, a *FinH* inhibitor, and EcoActive, an anti-*E.coli* bacteriophage, in patients with active CD (NCT03943446, NCT03808103) to know the potential of these emerging therapies.

In addition to AIEC, *Mycobacterium avium* subspecies, *Paratuberculosis*, *Pseudomonas aeruginosa*, and *Fusobacterium nucleatum* have also been reported as potential pathobionts in patients with CD [132,133,134]. Rather than acting against individual pathobionts, a combination of antibiotics including Clarithromycin, Rifabutin, and Clofazimine (Table 3) is also being evaluated for its potential effect in patients with CD [135].

## 5. Predictor Biomarkers for Evaluating Therapeutic Response to Different IBD Treatments

**Figure 3:**
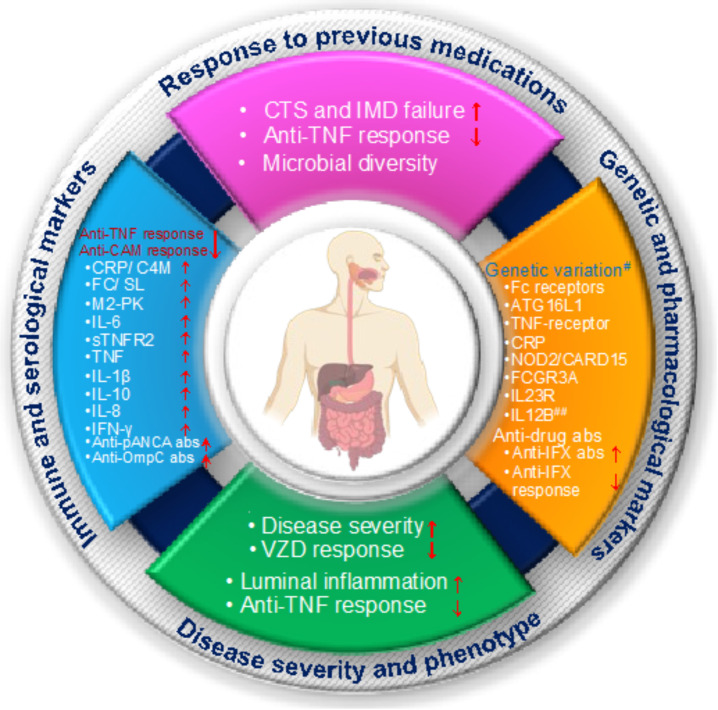
Predictive biomarkers for different IBD treatments. The figure shows the list of different predictive biomarkers that are associated with disease severity and response to clinical therapy in patients with IBD. ^#^ Genetic variations in these genetic markers could predict a non-responsiveness to anti-TNF (infliximab) therapy in IBD patients. ^##^ Heterozygous genotype of IL12B—10993 G > C (rs3212217) positively correlated with non-responsiveness to anti-TNF therapy in UC patients. CRP: C-reactive protein; FC: fecal calprotectin; SL: stool lactoferrin; CTS: corticosteroids; IMD: immunomodulators; IFX: infliximab; VZD: vedolizumab; TNF: tumor necrosis factor; C4M: Matrix metalloproteinases-mediated degradation of type IV collagens; IL: interleukin; sTNFR2: Serum soluble tumor necrosis factor receptor-2; IFN: Interferon; FCGR3A: Fc Gamma Receptor 3a; abs: antibodies; pANCA abs: perinuclear antineutrophil cytoplasmic antibodies; Anti-OmpC abs: anti- outer-membrane protein OmpC of *Escherichia coli* antibodies; Fc: fragment crystallizable; NOD: nucleotide-binding and oligomerization domain; CARD 15: caspase recruitment domain-containing protein 15; ↑: increase in levels; ↓: decrease in levels.

As discussed in previous sections and in STRIDE guidelines, the primary goal of IBD treatment is to provide symptomatic relief, promote endoscopic healing and prevent disease flare-up; thus, predicting response to IBD therapy is critical to avoiding severe IBD-related complications such as surgery and hospitalizations. Furthermore, because many IBD patients become intolerant or lose response to treatment over time, the ability to predict response to treatment allows for more personalized treatment options for patients [136] (Figure 3).

### 5.1. Biomarkers for Response to Aminosalicylates

Although 5-ASA therapy is the first line of treatment for mild-to-moderate UC patients, its association with an increased risk of treatment failure (17 to 75%) or disease relapse is a major concern in clinical management of patients [137,138,139,140,141,142]. Therefore, early identification of 5-ASA treatment failure is crucial to avoid disease progression; however, the lack of standardized parameters for treatment failure makes this difficult [143]. According to a multi-center prospective cohort study in 467 pediatric UC patients, a predictive model was developed, including an initial clinical activity and treatment response to Mesalazine at week 4, to predict the corticosteroid-free remission at 52 weeks [144]. This predictive model is based on several non-genetic and genetic factors, including 25(OH)D levels, rectal eosinophil counts (less than 32 per high power field), rectal gene expression, gut microbial dysbiosis, primarily *Clostridiales* depletion, ion channel gene down-regulation, and an abundance of antimicrobial peptides. Furthermore, several genetic markers, such as IBD patients with homozygous alleles for the IL23RG9T gene, demonstrated a better response [145], whereas IBD patients with the GC genotype in the Rac1 gene (rs34932801) demonstrated a lower response to Azathioprine therapy [146]. In contrast, IBD patients with *GSTM1* (glutathione S-transferase) gene deletion showed a poor response to treatment [147] and required treatment escalation to anti-TNF therapy [144].

### 5.2. Biomarkers for Response to Corticosteroids

Because corticosteroid treatment response has been highly variable and is associated with increased side effects, early prediction of treatment failure is critical for treatment escalation. A prospective cohort of 423 Chinese UC patients revealed that only 41.6% of patients respond to corticosteroid therapy for an extended period [148]. Further multivariate analysis of different risk factors identified multiple predictive markers such as Tenesmus as a negative predictor of corticosteroids response (OR = 0.336; 95%CI: 0.147–0.768; *p* = 0.013), and weight loss as a predictor of treatment failure (OR = 5.662; 95%CI: 1.111–28.857; *p* = 0.040) [148]. However, the baseline levels of FC and UCEIS show the best predictive correlation with the short-term clinical response to corticosteroids in acute severe UC patients [149]. Additionally, short-term response to corticosteroids also correlated well with long-term remission maintenance on 5-ASAs or immunomodulators [150,151].

### 5.3. Biomarkers for Response to Biological Treatments

Biologics have emerged as a highly promising approach to treating patients with severe IBD over the last two decades, however not all IBD patients respond well to the biological therapies [152]. Emerging clinical studies have reported that around 13–46% of IBD patients are non-responders or lost response to biologics within 12 weeks of therapy [152], implying that either pathological mechanisms that modulate GI inflammation differ between patients or that blocking a specific cytokine leads to the development of alternative compensatory pathways in the patients. As a result, the early identification of factors associated with clinical responses to biological therapies, such as immune markers, microbiome, anti-drug-antibody, and genetics, is critical for patients when selecting or monitoring biologics or combination therapy.

**Immune markers:** Immune markers such as fecal inflammatory markers (calprotectin and lactoferrin) and blood CRP are known predictors of active intestinal inflammation and long-term response to treatment in both CD and UC patients [153,154]. Higher levels of FC displayed an association with non-response to Infliximab in severe UC patients, and were an indication of treatment failure [155,156], whereas a lower level of FC (<250 µg/g), after eight weeks of initiation of Vedolizumab treatment in IBD patients, can positively predict a histological and endoscopic response to therapy [157]. Other emerging fecal inflammatory markers, such as the dimeric M2 isoform of pyruvate kinase (M2-PK), have been found to be more accurate in predicting response to Infliximab in patients with active UC [155] than non-specific FC. Furthermore, non-responders to anti-TNF and anti-integrin therapies show higher levels of IL-6, sTNFR2 e, TNF-α, IL-1, IL-10, IL-8, and IFN-γ than responders [158,159,160,161].

**Microbiome:** Although the etiology of IBD is unknown, the complex interaction of the gut microbial community with immune cells may influence disease severity and susceptibility to immune therapy in IBD patients. For example, higher abundance of *Bifidobacterium*, *Clostridium colinum, Eubacterium rectale*, uncultured *Clostridiales* and *Vibrio* and lower levels of *Streptococcus mitis* have been positively correlated with better response to anti-TNF therapy in IBD patients [162], while patients with gut microbial dysbiosis [163] or with additional fibro-stenotic disease showed a poor response rate to anti-TNF treatment and often required surgery to manage the disease [164,165,166,167]. In addition, a higher abundance of butyrate-producing species (such as *Roseburia inulinivorans* and *Burkholderiales*) and a higher synthesis level of branched-chain amino acids are shown to be a positive predictor of remission and the clinical response to Vedolizumab [168]. Although, given the diversity of changes in different populations and the lack of statistical power in studies, classifying microbial biomarkers for response to biological therapies appears to be a moving target.

**Anti-drug-antibody:** Some biological therapies can elicit an immune response with the consequent production of anti-drug antibodies (ADA), which in contrast can lead to loss of their responses in IBD patients [169,170]. For example, long-term therapy with Infliximab might stimulate anti-Infliximab antibodies, and cause increased risk of treatment failure, hence in case of >3 μg/mL Infliximab therapy, monitoring of serum ADA is crucial to ensure disease remission in IBD patients [171]. Furthermore, multiple studies have found a link between anti-neutrophil cytoplasmic antibodies (pANCA) and anti-OmpC (*Escherichia coli* outer membrane porin) antibodies and a poor response to Infliximab therapy. [172,173,174].

**Genetic markers:** Similarly, the genetic profiling of markers has shown a positive correlation with predictive response to biological treatment in IBD patients. Most genetic predictive markers are related to cytokines or their receptors and immunoglobulin receptors, including *TNF/TNF*-receptor genes, *ATG16L1* gene, apoptosis genes, *NOD2/CARD15* genes, *CRP, IL23R* and *IL12* genes and Fc receptors related genes [175,176,177,178]. For example, genetic variations in *TNF-β* and *TNFRSF1B* genes (rs1061624_A-rs3397_T) together with a minor allele (A) polymorphism of *TNF* gene (rs1800629) could predict a non-responsiveness to anti-TNF (infliximab) therapy in CD patients [179,180,181], while a heterozygous genotype of *IL12B—10993* G > C (rs3212217) is positively correlated with non-responsiveness to anti-TNF therapy in UC patients [182]. Similarly, an apoptosis related Fas ligand’s CC genotype positively correlated with non-response to infliximab, while TC or TT genotype predict response to anti-TNF therapy [179]. In addition, an association between the *FCGR3A* and *ATG16L1* gene polymorphism and response to anti-TNF treatment revealed a link between V/V allotype and decreased CRP levels in CD patients [176,177,183], whereas IBD patients with the ATG16L1 T/T and C/T genotypes had significantly higher CRP levels and showed a better response to Adalimumab than patients with the C/C genotype [175,184].

**Mucosal transcriptomics markers:** Biologics therapies can significantly modulate the expression level of mucosal cytokines and suppress the inflammation; therefore, a change in the transcript level cytokines can be used as predictive therapeutic biomarkers of their efficacy. For example, multiple studies have shown reduced mucosal TNF- α transcript levels in response to IFN therapy patients, which correlated well with disease remission and mucosal healing in both UC and CD patients [185,186]. Similarly, blood or mucosal transcript levels of several markers, such as IL-17A, IL-6, IL-7R and interferon (IFN)-γ have been explored as predictive therapeutic efficacy biomarkers of anti-TNF or anti-α4β7 therapies in CD and UC patients (Table 4) [187].

**MicroRNAs:** MicroRNAs (miRNAs) are small, non-coding RNAs and are known to be involved in gene expression and different cellular processes including inflammation [188]. Recently some studies have found a correlation between seven miRNAs levels and anti-TNF therapy responses (Table 4) [189,190], although these are preliminary results and need further investigations in larger, more diverse populations to explore their potential as predictive biomarkers.

**Proteomics markers:** Protein levels are probably the most ubiquitously affected profile in both serum and inflamed mucosa during disease, response and recovery; hence they are being rapidly explored as a potential diagnostic [191] and therapeutic response in IBD [192,193]. Recently D’Haens et al. [194] reported differential serum levels of 13 proteins (ANG1, ANG2, CRP, SAA1, IL-7, EMMPRIN, MMP1, MMP2, MMP3, MMP9, TGFA, CEACAM1, and VCAM1) in CD patients, which also correlated well with remission in CD patients and serum CRP. Similarly, some other studies have further explored the capacity of proteomics and identified several markers, such as platelet aggregation factor 4 [PF4], sCD40L, IL-6, apolipoprotein A-I, apolipoprotein E, complement C4-B, plasminogen, serotransferrin, beta-2-glycoprotein 1 and clusterin for predicting therapeutic response in IBD patients [193]. Although the proteomics markers offer an innovative approach for evaluating therapeutic responses in IBD patients, but inconsistency in markers signature across studies and lack of follow-up validation studies on larger cohorts of patients, hinders the identification of universal proteomics biomarker for predicting therapeutic response in IBD patients.

## 6. Future Directions

Both CD and UC are heterogeneous diseases and depend on multiple factors. Because of this they cannot justify a one-medicine-fits-all principle and therefore present a significant challenge to patients and clinicians. Although several new CD and UC therapies are promising in controlling acute diseases, they are largely ineffective in preventing spontaneous disease flare-ups or reversing disease states. In our view, targeting only one particular aspect of the disease may not have a significant outcome on the management of IBD; therefore, future strategies for IBD treatment should be directed to target multiple disease factors at a time and align with STRIDE-II recommendations to facilitate the long-term outcome of IBD. Although there are emerging reports of the use of combined biologic agents for refractory IBD with encouraging outcomes, highlighting the potential of combination therapies, there is still a huge unmet need for novel therapeutic options as many IBD patients do not respond to clinically approved drugs or loose response overtime.

Thus, a plethora of new therapeutic approaches are currently being evaluated in clinical trials for IBD but designing combinational therapy trials is a daunting task and it can be difficult to know which therapies to use and in which order as the therapeutic response may vary between individuals.

In this regard, advanced, sophisticated molecular tools, and animal models could help to predict the therapeutic response to potentially synergistic or antagonistic effects of combination. Efforts should be made to use advance metagenomics and computational techniques and strictly align the clinical trials end points with STRIDE-II recommendations, including mucosal healing on endoscopy, deep remission (clinical remission plus mucosal healing), and transmural healing. This can be further augmented by combining predictive microbial and immune signature profiles along with efficacy monitoring markers to select the best treat-to-target option or combinations and to guide treatment toward achieving the short- and long- term therapeutic goals of IBD management. Moreover, profiles of individual patient metabolomes could also be used to determine the optimal composition and diet for treatment. This can ultimately help us to further raise the bar for future drugs in IBD therapy and possibly reduce IBD-associated complications such as surgery. Nevertheless, if we achieve this, we can pave the way for a tailored therapy algorithm for every patient suffering from IBD and reduce the unnecessary burden of hospitalization.

## Figures and Tables

**Table 1:** Commonly used IBD activity indices to measure the disease severity.

CD and IBD-U Activity Indexes	UC Activity Indexes
Crohn’s Disease Activity index (CDAI) Uses a combination of five variables, including discharge, pain, restriction of sexual activity, type of perianal disease, and degree of induration. Simple index that is clinically used for patient management.	Ulcerative colitis disease activity index (UCDAI) Uses a combination of GIT symptoms, endoscopic appearance, and physician global assessment to access the disease activity in UC patients.
Pediatric Crohn’s Disease Activity index (PCDAI): Relies on clinical symptoms, anthropometric and serological biomarkers in pediatric CD patients Correlates poorly with endoscopic disease activity in newly diagnosed CD children	Pediatric Ulcerative Colitis Activity Index (PUCAI) Focuses mainly on clinical symptoms in pediatric UC patients. Correlates well with the endoscopic disease severity, however, significant variation in clinical symptoms may arise in children with inflamed colons
Weighted Pediatric Crohn’s Disease Activity index (wPCDAI) Uses a combination of clinical symptoms, physical examination, and serological biomarkers in pediatric CD patients and all variables are mathematically weighted to produce an overall score. Correlates poorly with endoscopic disease activity or mucosal healing CD children	Ulcerative Colitis Endoscopic Index of Severity (UCEIS) Uses a combination of clinical symptoms in pediatric UC patients to evaluate endoscopic severity, including vascular pattern, bleeding, erosions, and Ulcers. Correlates well with the disease severity and can be used in predicting therapeutic response in patients.
Harvey-Bradshaw index (HBI) or simple endoscopic score Associated with elevated CRP and thrombocytes. Not associated with the endoscopic activity	Mayo clinic score Uses a combination of clinical symptoms, endoscopy, aspects of quality of life and the physician’s global assessment (PGA) Shows good correlation with fecal calprotectin, C-reactive protein, and the erythrocyte sedimentation rate (ESR)
Mucosal Inflammation Non-invasive index (MINI): Uses a combination of clinical symptoms, serological markers, fecal calprotectin and the simple endoscopic score for Crohn’s disease (SESCD). Correlate with mucosal inflammation.	Simple Clinical Colitis Activity Index (SCCAI) Uses only the clinical symptoms. Shows moderate to strong correlation with endoscopic activity (Mayo endoscopic sub-score) Shows a good correlation with feacal calprotectin and CRP
The simple endoscopic score for CD (SES-CD) Uses a combination of endoscopic parameters including ulcer size, estimates of the ulcerated and affected surface, and the presence of luminal narrowing.	The Modified Baron Score Uses a combination of endoscopic variables including vascular pattern, granularity, hyperaemia, friability, ulceration, bleeding.
The magnetic resonance index of activity (MARIA) and the Clermont score Uses a combination of two useful MRI indices in assessing of the CD endoscopic ulcerations. Useful in assessing in therapeutic endpoints.	Novel integral disease index of UC activity (NIDI) or Yamamoto-Furusho Index Uses a combination of clinical, biochemical, endoscopic, and histologic biomarkers of UC patients to assess the disease activity. Provides more objective evaluation of disease activity using multiple variables.
The Lewis score (LS) and Capsule Endoscopy Crohn’s Disease Activity Index (CECDAI) Use a combination of two endoscopic scores used to evaluate the visualized images. Shows a better association with the active intestinal inflammation and high disease activity than LS.	UC Colonoscopic Index of Severity (UCC) Uses a combination of endoscopic parameters including vascular pattern, granularity, ulceration, bleeding, friability. Provides an accurate and simple scoring The Walmsley index Non-invasive index used to assess disease activity in adults with UC. Uses a combination of combination of clinical and laboratory markers including haemoglobin, haematocrit, platelet count, erythrocyte sedimentation rate, and serum albumin

CD: Crohn’s disease; IBD-U: inflammatory bowel disease unclassified; UC: ulcerative colitis; CRP: c-reactive protein; GIT: gastrointestinal tract; C-reactive protein (CRP).

**Table 2:** Therapeutic options for UC and CD.

Drug Name	Mechanism of Action	Route	Indications	Development Status
**Aminosalicylates** Balsalazide Mesalamine Olsalazine Sulfasalazine	* Anti-inflammatory CXY and LXY inhibitor * Anti-inflammatory Prostaglandins inhibitor	PO PO, rectal PO PO	Mild-to-mod UC Mild-to-mod UC UC UC	Approved Approved Approved Approved
**Corticosteroides** Budesonide Methylprednisolone Prednisolone Prednisone	GRs inhibitor Anti-inflammatory Anti-inflammatory Anti-inflammatory	PO PO, IV PO PO	Mild-to-mod CD, UC Mod-to-severe CD, UC Mod-to-severe CD, UC Mod-to-severe CD, UC	Approved Approved Approved Approved
**Immunomodulators** Azathioprine Cyclosporine Mercaptopurine Methotrexate Tacrolimus	Purine synthesis inhibitor T-cells inhibitor (IL-2) Purine synthesis inhibitor DHFR inhibitor Inhibits IL-2 transcription	PO PO, IV PO PO, SC PO, IV	CD, UC UC CD, UC Active CD Mod-to-severe CD, UC	Approved Approved Approved Approved Approved
**Antibiotics** Ciprofloxacin Metronidazole Vancomycin Rifaximin Amoxicillin/metronidazole/doxycycline/vancomycin Metronidazole + tobramycin	Topo and gyr inhibitor Bacterial DNA synthesis Cell wall synthesis inhibitor Protein synthesis inhibitor Cell wall synthesis inhibitor Bacterial DNA synthesis Protein synthesis inhibitor Cell wall synthesis inhibitor Bacterial DNA synthesis	PO, IV PO PO PO PO PO	Active CD and pouchitis Active CD and pouchitis Active CD Active CD Acute severe or chronic UC Acute severe UC	Approved Approved Approved Approved Approved Approved
**TNF-α inhibitors** Adalimumab Infliximab Certolizumab Golimumab	Anti-TNF-α ab (IgG1) Anti-TNF-α ab Anti-TNF-α ab Anti-TNF-α ab	SC SC, IV SC SC	CD, UC Mod-to-severe CD, UC Mod-to-severe CD Mod-to-severe UC (adult)	Approved Approved Approved Approved
**CAM inhibitors** Natalizumab Vedolizumab	Anti-α4β1-integrin Anti-α4β7-integrin	IV SC, IV	Mod-to-severe CD CD, UC	Approved Approved
**IL-12/-23 inhibitors** Ustekinumab	Anti-IL-12/IL-23 (p40) ab	IV	CD	Approved
**JAK inhibitors** Tofacitinib	Janus Kinase	PO	UC	Approved

* Specific MOA is not known but shows anti-inflammatory effect. Mab: Monoclonal antibody; CAM: Cell adhesion molecules inhibitors; MOA: Mechanism of Action; CXY: Cyclooxygenase; topo: DNA topoisomerase; gyr: DNA gyrase; LXY: lipoxygenase; GRs: intracellular glucocorticoid receptors; Mod: Moderate; DHFR: Dihydrofolate reductase.

**Table 3:** Emerging therapies for UC and CD.

Drug Name	Mechanism of Action	Route	Indication	Development Status
**Immunomodulators** Neihulizumab BBT-401	Activate T-cells Inhibits signalling pathways	IV PO	Mod-to-severe UC Active UC	Ph-II Ph-II
**Antibiotics** EB8018/TAK-018 EcoActive Ceftriaxone Clarithromycin + rifabutin + clofazimine Ciprofloxacin + Doxycycline + Hydroxychloroquine + Budesonide Azithromycin + Metronidazole Amoxicillin + metronidazole + doxycycline	FimH inhibitor Anti-*E. coli* bacteriophage Antibiotics Antibiotics Antibiotics Antibiotics Antibiotics	PO PO PO PO PO PO PO	Active CD Inactive CD UC CD CD CD UC	Ph-II Ph-II Ph-II Ph-II Ph-II Ph-III Ph-II
**TNF-α inhibitors** Golimumab PF-06480605 ABBV-323	Anti-TNF-α ab Anti-TNF-α ab CD40 antagonist	SC SC, IV SC, IV	Ped UC Mod-to-severe UC Mod-to-severe UC	Ph-III Ph-IIA Ph-II
**CAM inhibitors** Etrolizumab AJM300 Ontamalimab	α4ß7 and αEß7 α4 integrin receptor Anti-MADCAM1 ab	SC PO SC	CD/UC Active UC Mod-to-severe UC, CD	Ph-I/II Ph-III Ph-Ib
**IL-12/IL-23 inhibitors** JNJ-67864238 Guselkumab Risankizumab Brazikumab Mirikizumab	IL-23 antagonist Anti-IL-23 (p19) ab Anti-IL-23 (p19) ab Anti-IL-23 (p19) ab Anti-IL-23p (p19) ab	PO SC SC IV, SC SC	Mod-to-severe UC Mod-to-severe UC, CD Mod-to-severe UC, CD Mod-to-severe CD CD	Ph-II Ph-III Ph-II Ph-II Ph-II
**IL-22 inhibitors** UTTR1147A	IL-22 inhibitor	IV	CD/UC	Ph-II
**IL-36 inhibitors** Spesolimab	Anti-IL-36R ab	IV	Mod-to-severe UC, CD	Ph-II/III
**IL-6 inhibitors** PF-04236921	Anti-IL-6 ab	SC	Mod-to-severe CD	Ph-II
**JAK/TYK inhibitors** PF-06651600 PF-6700841 Upadacitinib BMS-986165 Filgotinib Itacitinib SHR-0302 TD-1473	JAK-3 inhibitor JAK-1/TYK2 inhibitor JAK-1 inhibitor TYK-2 JAK-1 inhibitor JAK-1 inhibitor JAK-1 inhibitor JAK inhibitor	PO PO PO PO PO PO PO PO	Mod-to-severe UC, CD Mod-to-severe UC, CD Mod-to-severe UC, CD Mod-to-severe UC, CD Mod-to-severe CD Mod-to-severe UC Mod-to-severe UC, CD Mod-to-severe UC, CD	Ph-II Ph-II Ph-II/III Ph-II Ph-II Ph-II Ph-II Ph-II/III
**Stem-cell therapies** Cx-601	Immune modulation	* IV	CD	Ph-III
**S1P inhibitors** Etrasimod Ozanimod	S1P receptor modulator S1P-1/5 receptor modulator	PO PO	Mod-to-severe CD, UC Mod-to-severe CD, UC	Ph-III Ph-III
**Antisense nucleotides** Mongersen	Immune modulation	PO	CD	Ph-III
IMU-838	Inhibit DHODH	PO	Mod-to-severe UC	Ph-II
**NKG** JNJ-64304500	Anti-NKG2D antibody	SC	Mod-to-severe CD	Ph-II
**FMT** SER-287	Probiotics (microbiome)	PO	Mild-to-moderate UC	Ph-1b

* IV: administered directly to the fistula site. DHODH: Dihydro-orotate dehydrogenase; S1P: Sphingosine-1-phosphate receptor; CAM: Cell adhesion molecule; MADCAM1: Monoclonal antibody that targets mucosal adhesion cell adhesion molecule; FMT: Fecal microbiota transplantation; Mod: Moderate.

**Table 4:** Putative biomarkers for evaluating anti-TNF therapeutic efficacy in IBD patients.

Biomarker	Anti-TNF Therapy: CD Patients		Anti-TNF Therapy: UC Patients	
	Expression in Responder	Expression in Mucosal Healing	Expression in Responder	Expression in Mucosal Healing
Mucosal transcripts TNF-α	↓	↓	↓	↓
IL-17A	↓	↓	↓	↓
IFN-γ	-	-	↓	↓
OSM	↓	-	↓	-
IL-7R ^#^	↓	-	↓	-
miRNAs	↓	-	↓	-
Proteomics	↓	-	-	-
Genomic	↓	-	↓	-

^#^ Reduced mucosal transcript levels of IL-7R also observed in responders to immunosuppressive/corticosteroid, anti-TNF, or anti-a4b7 therapies in both severe CD and UC patients. TNF-α: tumour necrosis factor-α; IFN-γ: interferon-γ; IL-17A: interleukin-17A; miRNAS: MicroRNAs; OSM: Oncostatin M; IL-7R: interleukin-7 receptor; ↓: decrease in expression; -: not known.

## Data Availability

Not applicable.

## Abbreviations

AMPK	Adenosine monophosphate-activated protein kinase enzyme
5-ASA	Aminosalicylates
BDP	Beclomethasone dipropionate
CS	Corticosteroids
IFX	Infliximab
VDZ	Vedolizumab
ADA	Adalimumab
USK	Ustekinumab
ETZ	Etrolizumab
FMT	Fecal microbiota transplantation
FC	Fecal calprotectin
